# Development of a Model of Crack Propagation in Multilayer Hard Coatings under Conditions of Stochastic Force Impact

**DOI:** 10.3390/ma14020260

**Published:** 2021-01-07

**Authors:** Alexey Vereschaka, Sergey Grigoriev, Anatoli Chigarev, Filipp Milovich, Nikolay Sitnikov, Nikolay Andreev, Catherine Sotova, Jury Bublikov

**Affiliations:** 1 Institute of Design and Technological Informatics of the Russian Academy of Sciences (IDTI RAS), 127994 Moscow, Russia; yubu@rambler.ru; 2 Department of Highly Effective Technologies and Processing (VTO), Moscow State Technological University STANKIN, 127994 Moscow, Russia; s.grigoriev@stankin.ru (S.G.); e.sotova@stankin.ru (C.S.); 3 Mechanical and Mathematics Faculty, Belarusian State University, 220050 Minsk, Belarus; chigarevanatoli@yandex.ru; 4 Materials Science and Metallurgy Shared Use Research and Development Center, National University of Science and Technology MISiS, 119049 Moscow, Russia; filippmilovich@mail.ru (F.M.); andreevn.misa@gmail.com (N.A.); 5 Department of Solid State Physics and Nanosystems, National Research Nuclear University MEPhI, 115409 Moscow, Russia; sitnikov_nikolay@mail.ru

**Keywords:** Markov processes, multilayer coatings, crack formation model, failure probability density function

## Abstract

The article deals with the problems of cracking in the structure of multilayered coatings under the conditions of stochastic loading process. A mathematical model has been proposed in order to predict the crack propagation velocity in the coating while taking the influence of interlayer interfaces into account. A technique for calculating the probability density distribution of the coating fracture (failure rate) has been developed. The probability of a change in the crack growth direction is compared with the experimental data that were obtained as a result of the studies focused on the pattern of cracking in the Zr,Nb-(Zr,Nb)N-(Zr,Nb,Al)N and Ti-TiN-(Ti,Cr,Al)N coatings under the conditions of the real stochastic loading of cutting tools during the turning. The influence of the crystalline structure of the coating on the cracking pattern has been studied. The investigation has found the significant effect of the crystalline structure of the coating layers on the cracking pattern.

## 1. Introduction

Brittle fracture is a crucial and often dominant factor that reduces the efficiency of wear-resistant coatings and cutting tools [1]. In general, the coatings are very hard and rather brittle coverings with a high tendency to crack formation [2,3]. Accordingly, the prediction of the crack formation in coatings and creation of the coatings with enhanced resistance to cracking is an important scientific and practical challenge. It is necessary to consider the fact that the cutting of materials is a complex stochastic process, which combines the influence of force and temperature factors, as well as oxidation, diffusion, and other processes. To date, there is no model that is able to take the effect of all the above factors into account, and it is hardly possible to create such a global model. Meanwhile, some models have been developed, and researchers continue to develop models that consider the process of coating functioning from different points of view, while using various modeling techniques. A finite element model (FEM) is quite often applied in order to simulate crack formation. In particular, in [4], brittle coating on an elastic substrate in four-point bending is investigated. The studies considered the influence of such factors, as random strength distribution, coating thickness, residual stresses, and coating modulus. The influence on the crack formation of such parameters, such as the number of load cycles, surface geometry, and properties of the coating material, was also investigated in [5]. A number of studies [6,7,8] used FEM in order to simulate crack formation in a coating under the influence of an indenter or a scratch tester pressed into the coating. During the scratch testing, a crack is formed in a surface part of the coating as a result of bending and tensioning, and then the crack develops and cuts the coating through [9]. FEM was also applied in order to study an influence of substrate surface roughness on stress distribution and following the delamination of the coating and substrate [10]. During the modeling using FEM, it has been found [11] that defects in a coating structure can generate stress peaks and high levels of deformation, which can lead to the formation of cracks and coating fracture. In [12], a coating is being modeled as an elastic layer, ideally connected to an elastic substrate, with a forming microcrack, which is assumed to arise upon contact with an indenter, because of high tensile stress. A profile of a propagating crack is being predicted depending on the coefficient of friction, fracture toughness, and sliding displacement. A shear lag model is considered in [13] for the analysis and measurement of shear strength at the “brittle coating–ductile substrate” interface. In particular, it is found that stresses in the coating plane are inhomogeneous and vary over the coating thickness. Consequently, a new criterion—Thickness-averaged In-plane Stress (TIS)—was proposed for assessing the phase-to-phase shear strength. The Discrete Element Method (DEM) is also used to predict the development of cracks [14]. In [15], various approaches were chosen to predict the formation of cracks during the scribing of a coating. In particular, papers considered the prediction of preferential failure mode, prediction of the critical load for cone crack formation by Lawn, and prediction based on Bower and Fleck’s equation and Hamilton’s equation. In order to predict the coating fracture pattern, a fracture toughness model, based on the microcrack formation theory, was also applied [16]. In this model, fracture toughness of the “coating/substrate” system is determined through the use of crack tip opening displacement (CTOD) [10] for measuring the overall growth of the microcrack in the direction of tension, while the dislocation movement relates to the propagation of the crack. In [17,18], it is proposed to use the technique of linear elastic fracture mechanics (LEFM) in order to predict the crack resistance of coatings. The crack spacing in the coating is determined based on geometry, material properties, and external loads. It has been found that the thicker and stiffer the coating is, the easier cracks can develop in it. Meanwhile, according to another paper [19], thicker coatings demonstrate less intense cracking. Internal defects of the coating, in particular, microdroplets, embedded in the coating structure, stimulate an increase and development of cracks [20,21,22,23,24].

It should be taken into account that the cutting process is stochastic and, accordingly, the force and temperature factors affecting coated tools are also stochastic in nature. Probabilistic approaches, considering the stochastic nature of the deposition process, are increasingly used for modeling the deposition of coatings [25,26,27,28,29,30,31]. It is also advisable to use the probabilistic approaches to model the process of coating fracture as a result of cracking.

Alternatively, in recent years, multilayer coatings, including those with nanometric layer thicknesses, have become increasingly widespread. The process of crack propagation in such coatings has a number of typical features [2,3,21] to be taken into account during the modeling. As a result of the simultaneous FEM modeling and the studies with nano-impact tests, it has been found that multilayer coatings have better resistance to crack propagation when compared to single-layer ones [32]. Similar results were also obtained in [33,34,35,36]. In [37], the studies have also revealed that, in multilayer coatings, the crack propagation is decelerated, due to the dissipation of energy by crack deflection along interfaces. The mechanisms of crack deceleration in the coatings with nanolayer structure were considered in [33,34,35,36]. In particular, tests detected the influence of interlayer interfaces on the deceleration of crack propagation due to their transformation into interlayer delamination or a change in the direction of crack development. A coating structure with alternation of harder and more ductile nanolayers provides an optimal combination of high resistance to wear and brittle fracture, according to the studies.

The conditions of stochastic loading typical for the zone of elastic contact between the chips and the tool rake face are considered [38,39,40]. During the cutting, the tool material is being affected by stochastically influencing force factors. As is known, the external sliding friction acts in the zone of elastic contact, and the stochastic friction force between the chips and the tool rake face determines the resistance to the movement of the chips [39,40]. The tangential contact stresses in the section of the elastic contact gradually decrease, while the distance from the cutting edge increases. During the cutting process, the friction process in the zone of elastic contact is determined by overcoming intermolecular bonds, that is, the adhesive interaction. The considered area is characterized by the influence of the stochastic tangential contact stresses in combination with the effect of high temperature, and both of these factors gradually decrease while the distance from the cutting edge grows.

The analysis of the papers published earlier finds that the proposed models of crack propagation in the coatings cannot be considered to be fully consistent with the real operation conditions, since the models do not take the influence of the following key factors into account:

- stochastic nature of the loading process, and
- multilayered structure of the coating, in which adjacent layers have different physical and mechanical properties, and interlayer interfaces have significant influence on the cracking pattern.

Thus, the task of this paper was to develop a mathematical model for predicting the development of a crack in a multilayer coating under the conditions of a stochastic load process. Because of the complex challenge to take into account all of the conditions of the cutting process in a single mathematical model, this paper only considers the effect of force factors. No factor influencing temperature, oxidation, and diffusion processes were taken into account. To make the modeling process easier, the task was divided into two stages, as follows:

- predicting the time during which a crack passes a layer of a multilayer coating, and
- predicting a change in the direction of crack development, while a crack passes an interface between two coating layers with different physical and mechanical properties.

As is known, successive alternation of nanolayers with identical properties forms the nanolayer structure of the coating. Thus, it will be possible to easily predict the crack propagation in a nanolayer structure with any number of layers after overcoming the challenge of predicting the development of a crack in a two-layer or three-layer system.

## 2. Materials and Methods

Coatings of Ti-TiN-(Ti,Cr,Al)N and Zr,Nb-(Zr,Nb)N-(Zr,Nb,Al)N were deposited while using the technology of filtered cathodic vacuum arc deposition (FCVAD) [41,42,43,44,45,46,47,48,49,50]. A VIT-2 unit (IDTI RAS–MSTU STANKIN, Moscow, Russia) was used to deposit the coatings, with two arc evaporators, generating a pulsed magnetic field, and one arc evaporator with filtering of the vapor-ion flow. Apart from the above, the system also included a source of pulsed bias voltage supply to a substrate, a dynamic gas mixing system for reaction gases, a system for automatic control of pressure in the chamber, and a process temperature control system, and a system for stepless adjustment of planetary gear rotation [41,42,43,44,45,46,47,48,49,50]. According to a number of papers [2,3,21,35,36,51,52,53], due to nanolayer structure, the coating demonstrates high hardness at elevated temperatures and high oxidation resistance. It was assumed that the coating had a three-layer structure [35,38,51,52,53,54,55,56,57,58,59].

An analysis of the microstructure and nanostructure of the samples was studied while using JEM 2100 (JEOL, Akishima, Japan) high-resolution (HR) transmission electron microscope (TEM) with an accelerating voltage of 200 kV. An FEI Quanta 600 FEG (Thermo Fisher Scientific, Waltham, MA, USA) scanning electron microscope (SEM) was also used to study the coating microstructure.

The mechanical and tribological properties of the Ti-TiN-(Ti,Cr,Al)N coating were investigated in our previous works [54,60].

## 3. Theoretical Background

### 3.1. Passage of a Crack through a Coating Layer

**Figure 1:**
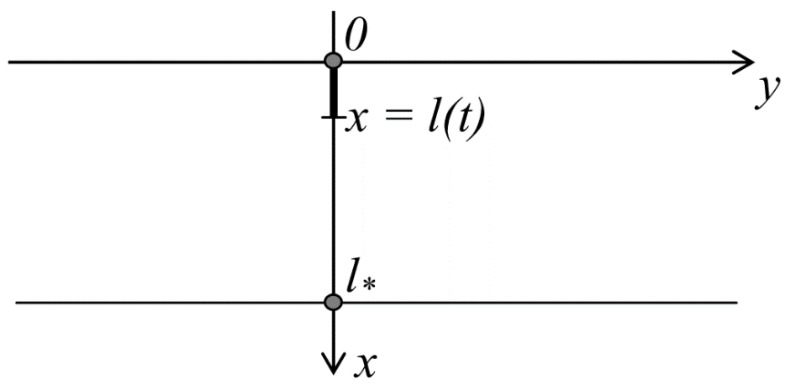
Coordinate system and model of the coating layer on the *0xy* plane, with thickness of l*.

A coating layer on the *0xy* plane, with thickness of l* (Figure 1) was considered.

A crack develops along the x axis, depending on the time point t or the number of cycles, and it is a random function in general case. At the time point *t*, the crack tip is at the point *x* = *l*(*t*). At each time point, the crack either retains its state or develops along the x axis. Reverse motion and possible deflection of the crack is not considered. Thus, the state of the crack at the time point *t* + Δ*t* is either *l*(*t* + Δ*t*) or *l* + Δ*l*, into which the crack tip passes with the probabilities:

(1) α(l)=P(l(t+Δt)=l)1−α(l)=P(l(t+Δt)=l+Δl)

Let $\Pi \left(l,t|l_{0},t_{0}\right)$ be a conditional probability that the crack tip is within the interval of (l,l+Δl) at the time point *t*, if at the time point *t*_0_, the crack tip is at the point *l*_0_. Let us assume that the process of the transformation of the crack tip from the given state into the next one only depends on this state and does not depend on the previous one (the history of the process), then the theory of Markov processes can be applied in order to describe the crack formation process, according to which all probabilistic information about the process (multivariate distribution) is expressed through the conditional probabilities $\Pi \left(l,t|l_{0},t_{0}\right)$, which satisfy the following equation:

(2) $$\Pi \left(l,t|l_{0},t_{0}\right)=\Pi \left(l,t-\Delta t|l_{0},t_{0}\right)\times \left(1-\alpha \left(l\right)\right)+\Pi \left(l-\Delta l,t-\Delta t|l_{0},t_{0}\right)\times \alpha \left(l-\Delta l\right)$$

Let the mean expected value for the conditional increment of the crack tip coordinate (at the fixed *l*(*t*)) in the short time Δ*t* be denoted through:

(3) $$m_{l}\left(\Delta t\right)=\langle l\left(t+\Delta t\right)-l\left(t\right)|l\left(t\right)\rangle$$

Accordingly, the dispersion (spread) of the above increment is denoted by:

(4) $$\sigma_{l}^{2}\left(\Delta t\right)=\;\langle {\left[l\left(t+\Delta t\right)-l\left(t\right)\right]}^{2}|l_{\left(t\right)}^{2}-m_{l}^{2}\left(t\right)\rangle$$

The limiting values (3), (4) are denoted, respectively:

Through the calculation of the mean value (3) of the conditional increment of the crack tip coordinate over the time *t*, we obtain:

(5) $$m_{l}\left(\Delta t\right)=\alpha \left(l\right)\Delta l$$

For dispersion, respectively:

(6) $$\sigma_{l}^{2}\left(\Delta t\right)=\left\{\alpha \left(l\right)-\alpha^{2}\left(l\right)\right\}{\left(\Delta l\right)}^{2}$$

Subsequently

(7) $$A\left(l\right)=\underset{\Delta t,\Delta l\to 0}{\lim}\alpha \left(l\right)\frac{\Delta l}{\Delta t}$$

(8) $$B\left(l\right)=\underset{\Delta t,\Delta l\to 0}{\lim}\left\{\alpha \left(l\right)-\alpha {\left(l\right)}^{2}\right\}\frac{{\left(\Delta l\right)}^{2}}{\Delta t}$$

Let us assume that ${\left(\Delta l\right)}^{2}=c\Delta t$

Afterwards

(9) $$\alpha \left(l\right)=\frac{B\left(l\right)+A\left(l\right)\Delta l}{2c}\;B\left(l\right)=A\left(l\right)\Delta l$$

For the random function of *l*(*t*) with the calculated mean *A* and the dispersion *B*, it is possible to get a differential equation for the probability $\Pi \left(l,t|l_{0},t_{0}\right)$. This is a Fokker–Planck–Kolmogorov equation.

(10) $$\frac{\partial \Pi }{\partial t}=-\frac{\partial }{\partial l}\left(A\left(l\right)\Pi \right)+\frac{1}{2}\frac{\partial^{2}}{\partial l^{2}}\left(B\left(l\right)\Pi \right)$$

The Equation (10) is a parabolic-type equation of the mathematical physics, for the solution of which the analytical and numerical methods are used. A discrete analogue of the crack propagation can be obtained if it is assumed that the time t and length l are quantized in time and states. The time takes on the values of *t* = 0, 1, 2, …n, *l* = *j* = 0, 1, 2, …*n*, *l*_0_ = *i* = 0, 1, 2, …*n*.

Subsequently, a replacement can be made: $\Pi \left(l,t|l_{0},t_{0}\right)=\Pi_{ij}\left(m\right),\;m=t-t_{0}$. A discrete equation of Markov type, which $\Pi_{ij}$ satisfies, has a form, as follows:

(11) $$\Pi_{ij}\left(m\right)=\alpha_{0-1}\Pi_{j,j-1}\left(m-1\right)+\left(1-\alpha_{j}\right)\Pi_{ij}\left(m-1\right)$$

Here, $\Pi_{ij}\left(m\right)$ is the probability of transformation from the state *i* to the state *j* in *m* steps.

It is clear that *i*, *j* are the discrete values of the crack length, *m* is the number of cycles, during which a crack develops from the length *i* to the length *j*.

In the theory of Markov processes, Equation (10) is called a direct Fokker–Planck–Kolmogorov (FPK) equation. An inverse FPK equation can be obtained in a similar way:

(12) $$-\frac{\partial }{\partial t_{0}}\Pi \left(l,t|l_{0},t_{0}\right)=A\left(l_{0},t_{0}\right)\frac{\partial \Pi \left(l,t|l_{0},t_{0}\right)}{\partial l_{0}}+\frac{1}{2}B\left(l_{0},t_{0}\right)\frac{\partial^{2}\Pi \left(l,t|l_{0},t_{0}\right)}{\partial l_{0}}$$

The difference between the Equations (10) and (12) is that, in (10), the right-hand side of the equation contains the differentiation with respect to *l*, while, in (12), the differentiation with respect to *l*_0_.

The process of solving an applied problem involves equations of both types, depending on the formulation of the problem. When it is required to define the probability that a crack tip reaches the length $l_{*}$ at the time point $T_{*}$, it is advisable to use an inverse equation, which takes the influence of the initial conditions into account. It is advisable to use the direct equation if at the initial time, the conditions are determined in a probabilistic manner, i.e., the crack initiation phase *l_0_* is a random variable. For the Equations (10) and (12), the initial condition is set for the deterministic initial crack length *l*_0_:

(13) $$\Pi \left(l,t|l_{0},t_{0}\right)=\delta \left(l-l_{0}\right)$$

The condition for non-negativeness of the probability is as follows:

(14) $$\Pi \left(l,t|l_{0},t_{0}\right)\geq 0$$

The condition for normalization of the probability is:

(15) $$\int \Pi \left(l,t|l_{0},t_{0}\right)\partial l=1$$

The boundary conditions can be different, depending on a specific problem to be solved.

If the crack length *l*(*t*) at the time point *t*_0_ is not constant and it is a random variable with the distribution density *P*_0_(*l*), then the condition is as follows:

(16) $$P\left(l,\;t_{0}\right)=\;P_{0}\left(l\right)$$

The probability densities should be consistent, i.e., they should satisfy the following equation:

(17) $$P\left(l,\;t\right)=\;\int_{-\infty }^{\infty }P\left(l_{0},t_{0}\right)\Pi \left(l,t|l_{0},t_{0}\right)dl_{0}$$

$\Pi \left(l,t|l_{0},t_{0}\right)\;$, makes it easier to solve the problem mathematically and, therefore, let us formulate the basic relations for finding *P*(*l*,*t*), taking into account (17).

There are different algorithms for finding *P*(*l*,*t*), depending on the problem to be solved.

At first, a functional solution to the Equation (12) with the initial condition (13) can be found, and then the Equation (17) can be solved.

Alternatively, an FPK equation can be directly obtained for *P*(*l*,*t*):

(18) $$\frac{\partial P\left(l,t\right)}{\partial t}=-\frac{\partial }{\partial l}\left(A\left(l,t\right)P\left(l,t\right)\right)+\frac{1}{2}\frac{\partial^{2}}{\partial l^{2}}\left(B\left(l,t\right)P\left(l,t\right)\right)$$

Additionally, the initial and boundary conditions can be formulated for it.

The initial conditions are as follows:

(19) $$P\left(l,\;t\right)\geq 0\;\;\;\;;\;\;\int_{-\infty }^{\infty }P\left(l,t\right)dl=1$$

**Figure 2:**
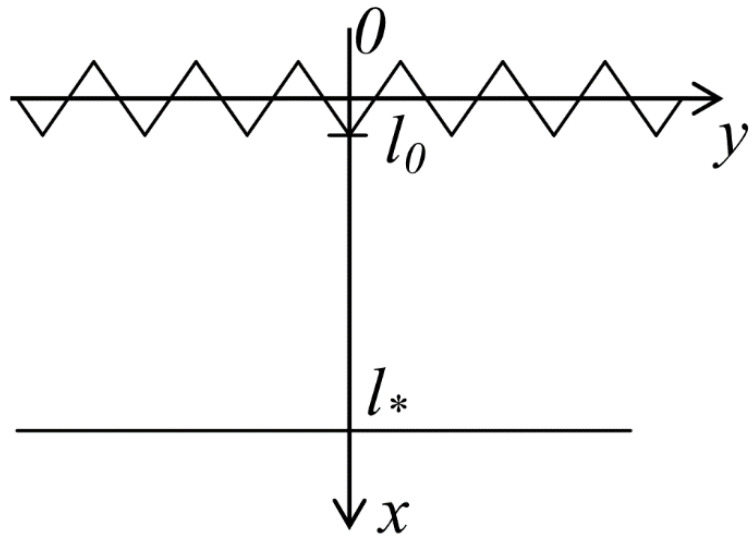
Diagram depicting the microcrack growth.

Let us consider a model of a rough upper layer and assume that the interface *x = 0* is an average surface, while a real surface has microroughness, where the value is determined by the random variable *l*_0_, with $0<l_{0}\ll l_{*}$ (Figure 2).

The growth of a crack starts from the length *l*_0_ (the length of the microcrack), taking the micro roughness into account.

The initial condition for the probability density has the form, as follows:

(20) $$P\left(l,\;0\right)=P_{0}\left(l\right)=\delta \left(l-l_{0}\right),\;l_{0}\in \left(0,l_{*}\right)$$

Let $F_{0,l}\left(t,l_{0}\right)$ be the probability that the microcrack with the initial value of *l_0_* reaches the layer interface during the time period t>0: either *l* = *0* or $l=l_{*}$. The case of reaching *l =* 0 corresponds to the motion of the crack tip towards the free surface, i.e., spallation, and the case of $l=l_{*}$ exhibits the through fracture by the crack of the upper layer with the thickness of $l_{*}$.

A case when the crack does not reach the layer interface, which is, the crack is damping in a layer, is determined by the probability of non-fracture of the layer.

(21) $$Q_{0,l_{*}}\left(t,l_{0}\right)=1-F_{0,l_{*}}\left(l,t_{0}\right)=P\left\{l_{0}<l\left(t\right)<l_{*},0<t<T\right\},\;l_{0}\in \left(0,l_{*}\right)$$

where $T\left(0,l_{0},l_{*}\right)$ is a random moment when the crack reaches the interface $l_{*}$, when the mean (expected value) and dispersions are constant: $A=A\left(l_{0}\right),\;B=B\left(l_{0}\right)$, the equation for $F_{0,l_{*}}\left(l,t_{0}\right)$ has the form, as follows:

(22) $$\frac{\partial F_{0,l_{*}}\left(t,l_{0}\right)}{\partial t}=A\left(l_{0}\right)\frac{\partial F_{0,l_{*}}\left(t,l_{0}\right)}{\partial l_{0}}+\frac{1}{2}B\left(l_{0}\right)\frac{\partial^{2}F_{0,l_{*}}\left(t,l_{0}\right)}{\partial l_{0}^{2}}$$

Let us consider a model for which a crack develops only rectilinearly into the depth of a layer, without spallation and deviation in the layer. In this case, the initial and boundary conditions are as follows:

(23) $$F_{0,l_{*}}\left(0,l_{0}\right)=1,\;\;\;\;\;\;\;\;\;\;\;\;0<l_{0}<d\;F_{0,l_{*}}\left(T,0\right)=0,\;\;\;\;\;\;\;\;\;\;\;\;F_{0,l_{*}}\left(T,l_{*}\right)=1$$

The following condition should be satisfied:

(24) $$\underset{t\to \infty }{\lim}F_{0,l_{*}}(t,l_{0})=1,\;(0<l_{0}\ll l_{*})$$

Let us denote the stationary approximation of the solution of Equation (22) on reaching by the crack tip of the interface *l* = l* through $F_{l_{*}}\left(l_{0}\right)$. The condition (24) means that the probability of the crack tip reaching the state of l* from the state of *l*_0_ is, in practice, a persistent event.

The stationary approximation of $F_{l_{*}}\left(l_{0}\right)$ satisfies the equation that follows from (22) at $\frac{\partial F_{0,l_{*}}}{\partial t}=0$.

(25) $$\frac{1}{2}B\left(l_{0}\right)\frac{\partial^{2}F_{l_{*}}\left(l_{0}\right)}{\partial l_{0}^{2}}+A\left(l_{0}\right)\frac{dF_{l_{*}}\left(l_{0}\right)}{dl_{0}}=0$$

Under the boundary conditions of

(26) $$F_{l_{*}}\left(0\right)=0,\;\;\;\;\;\;\;\;\;\;\;\;\;F_{l_{*}}\left(l_{*}\right)=\;1$$

the solution of the Equation (25) under the condition (26) in the analytical form is as follows:

(27) $$F_{l_{*}}\left(l_{0}\right)=\frac{\int_{l_{*}}^{l_{0}}exp\left[-\varphi \left(x\right)\right]dx}{\int_{l_{*}}^{0}exp\left[-\varphi \left(x\right)\right]dx}$$

(28) $$\varphi \left(x\right)=\int \frac{2A\left(x\right)}{B\left(x\right)}dx$$

It is clear that

(29) $$F_{l_{*}}\left(l_{0}\right)<1\;\mathrm{at}\;0<l_{0}\ll l_{*}$$

In order to further specify the problem, it is necessary to know the mean (expected value) *A*(*x*) and the *B*(*x*), a dispersion of the random function *l*(*t*), where the probability density satisfies the Fokker–Planck–Kolmogorov equations. As a rule, in this case, the *l*(*t*) satisfies the stochastic differential equation:

(30) $$\begin{array}{c} \frac{dl}{dt}=f\left(l,t\right)+g\left(l,t\right)n\left(t\right)\\ l\left(t_{0}\right)=l_{0} \end{array}$$

(31) $$\langle n\left(t\right)\rangle =0,\;\langle n\left(t_{2}\right)n\left(t_{1})\right\rangle =\frac{N_{0}}{2}\delta \left(t_{2}-t_{1}\right)$$

Other forms of the Equation (30) are as follows:

(32) dl=f(l,t)dt+g(l,t)dv(t)

(33) $$l\left(t\right)=l\left(t_{0}\right)+\int_{t_{0}}^{t}f\left(l\left(t\right),t\right)dt+\int_{t_{0}}^{t}g\left(l\left(t\right),t\right)dv\left(t\right)$$

The presented formulas make it possible to use Markov models of random processes to describe the processes of fatigue fracture that occur due to crack development.

The Paris’ law, based on the fact that all events on a crack tip, including the crack propagation velocity, depend on the stress intensity factor, can be written as:

(34) $$\frac{dl}{dn}=f\left(\Delta K\right)$$

where *n* is the number of load cycles,

$\Delta K=K_{max}-K_{min}$, *K* is the stress intensity factor.

While addressing the time dependence, let us denote n·τ=t, where *τ* is the cycle time, then the Equation (34) can be presented as:

(35) $$\frac{dl}{dt}=\frac{f\left(\Delta K\right)}{\tau }=f_{1}\left(\Delta K\right)$$

The Equation (35) describes a deterministic process of the crack growth. However, in fact, it is a stochastic process, in which only the average crack propagation velocity can be determined, with an error of *n*(*t*), where the average value is ⟨n(t)⟩=0. Subsequently, the Equation (35) can be randomized and, as a result, after adding a random error *n*(*t*) to the right-hand side of the Equation (35):

(36) $$\frac{dl}{dt}=f_{1}\left(\Delta k\right)+\frac{N_{0}}{2}n\left(t\right)$$

### 3.2. Calculation of Coating Reliability Based on a Model with Independently (Sequentially) Fracturing Layers

The state of each layer does not depend on the states of other layers. A fracture of the upper layer leads to the failure of the entire coating. Let *Qς*(*t*) be the probability of the operational condition of the entire coating at the time point *t* and *T_i_*–the period of time during which an *i*th layer stays not damaged (a random variable). Afterwards,

(37) $$Q_{\varsigma }\left(t\right)=P\left[T_{1}>t\cap T_{1}+T_{2}>t\cap T_{1}+T_{2}+T_{3}>t\right]$$

The probability of simultaneous event written in brackets for independent events is:

(38) $$Q_{\varsigma }\left(t\right)=P\left(T_{1}+T_{2}>t\right)P(T_{1}+T_{2}+T_{3}>t)$$

By denoting

(39) $$Q_{j}\left(t\right)=P(\underset{j}{\sum }T_{j}>t)$$

we obtain

(40) $$Q_{\varsigma }\left(t\right)=\underset{i}{\sum }Q_{i}\left(t\right)$$

The probability of the coating fracture is:

(41) $$F_{\varsigma }\left(t\right)=1-Q_{\varsigma }\left(t\right)$$

The probability density (failure rate) is introduced by the formula:

(42) $$P_{\varsigma }\left(t\right)=-\frac{dlnQ_{\varsigma }\left(t\right)}{dt}=\underset{i}{\sum }P_{i}\left(t\right)$$

In a case when

(43) $$P_{i}\left(t\right)=\lambda_{i}-const$$

where *λ_i_* is the fracture rate.

Subsequently, the average time until the fracture of the three-layer structure is:

(44) $$T_{\varsigma }=\frac{1}{\sum \lambda_{i}}$$

**Figure 3:**
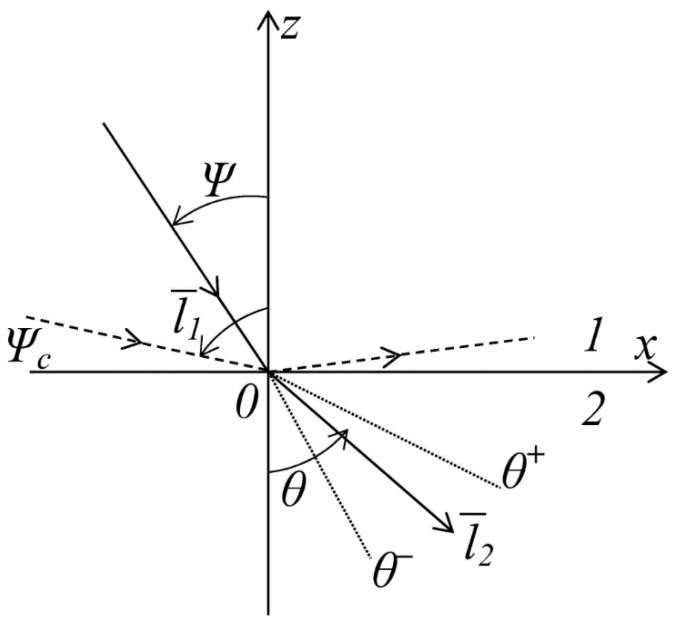
Diagram of a model presenting the crack passing through the layer interface.

Two layers, which were rigidly connected to each other, were considered, with the different physical and mechanical characteristics of $G_{i},\;v_{i},\;\rho_{i},\;i=1.2$ (shear modulus, Poisson’s ratio, density) (Figure 3).

The crack trajectory (Material 1) is curvilinear in a real medium, and it fluctuates near the x axis. The average time of the layer fracture depends on the crack length. The minimum time of the layer fracture will be obtained for a crack rectilinear in average, and this is the lowest estimate of the crack resistance of the layer. The maximum time of crack resistance will be obtained in the case when the crack trajectory significantly deflects from the straight line (for example, it is a fractal curve with a dimension over 1). As is known, the crack propagation is the process of transformation of the deformation energy into the bond-breaking power, i.e., the formation of a free surface [31]. Thus, the crack trajectory is a line (surface), with a density vector of the elastic energy flux (Umov–Poynting vector) that is directed along it. The optical–mechanical analogy can be applied here. Let us consider the transmission of energy through the layer interface, namely, a change in the direction of the energy density vector. Let us introduce the transmission coefficient T and the energy reflections at the layer interface. Let $P_{1}$ be the power that is supplied with the crack from Layer 1 to the layer interface, $P_{2}$–the power, passing into Layer 2, and $P_{11}$–the power, which did not pass into Layer 2 through the layer interface, and it is localized at the layer interface, then

(45) $$R=\frac{P_{11}}{P_{1}},\;\;\;T=1-\frac{P_{11}}{P_{1}}$$

Let assume that the directing unit vector of power density $\bar{e_{1}}$ forms the angle *Ψ* with the axis *z* in Layer 1, and the vector $\bar{e_{2}}$ forms the angle θ with the axis *z* in Layer 2.

It is clear that $\left|\bar{e_{1}}\right|=\left|\bar{e_{1}}\right|=1$.

A Snel van Royen formula (used in optics and acoustics) can be written, depending on the ratio of the physical and mechanical characteristics of the layer.

(46) n1sinΨ=n2sinθ,  ni=CiC0,  Ci=Giρi,       C0=G0ρ0

where $G_{i}$ is the shear modulus of the *i*th layer material and $\rho_{i}$ is the density of the *i*th layer material.

In general, the angle *θ* determines the initial direction of the crack in Layer 2. A special case corresponds to the scenario of the fracture of the layer interface (delamination). The situation takes place when $\Psi =\Psi_{c}$, a critical angle.

In this case, the energy is localized around the interface between the layers.

(47) Ψc=arcsin{n2n1}=arccos{1−n22n12}12

The power transmission coefficient *T* is expressed in terms of the $\Psi ,\;\Psi_{c}$ angles.

(48) T=4 sinΨ(sin2Ψ−sin2Ψc)12{sinΨ+(sin2Ψ−sin2Ψc)12}2sinΨc={1−n22n12}12

In the case of a crack, close to a vertical one, $\Psi =\theta =\frac{\pi }{2}$.

(49) $$T\approx \frac{4n_{2}n_{1}}{{\left(n_{1}+n_{2}\right)}^{2}}$$

When the crack tip in Layer 1 approaches the interface layer at the angle of $\Psi =\Psi_{c}$, close to the critical one, then

(50) $$T\approx 4\left\{1-\frac{\Psi_{c}^{2}}{\Psi^{2}}\right\},\;\;\;\Psi \geq \Psi_{c}$$

In general case, the Umov–Poynting vector is described for the elastic layers through the following formula:

(51) $$P_{j}=-\sigma_{ij}\dot{u_{i}},\;\;\;\;\;\;i,j=1,2,3$$

where $\sigma_{ij}$ is the stress tensor, $u_{i}$ are the components of the displacement velocity vector, and $P_{j}$ are the components of the vector $\bar{P}$.

The angle under which the crack from Medium 1 enters the interface with Medium 2 is not known, and it can be assumed that the angle of incidence is random. Accordingly, the angle under which the crack enters Medium 2 is also a random angle, due to the connection between these angles under the Snel van Royen law. Therefore, the process of the crack passing from Layer 1 into Layer 2 will be characterized by the conditional probability density $f\left(t_{2},\theta_{2}\;|t_{1},\;\theta_{1}\right)$, which satisfies the equation, as follows:

(52) $$\frac{\partial f\left(t_{1},\theta \;|t_{0},\;\theta_{0}\right)}{\partial t}=\frac{B}{2\sin\theta }\frac{\partial }{\partial \theta }\left(\sin\theta \frac{\partial f}{\partial \theta }\right)$$

The following expression should be taken as an initial condition:

(53) $$f{|}_{t=t_{0}}=\frac{\delta \left(\theta -\theta_{0}\right)}{2\pi \sin\left(\theta -\theta_{0}\right)}$$

where δ(θ) is the Dirac function.

With the initial condition (53), the solution of the Equation (52) is presented as:

(54) $$f\left(\theta |t-t_{0},0\right)=\frac{1}{4\pi }\overset{\infty }{\underset{n=0}{\sum }}\left(2n+1\right)P_{n}\left(\cos\theta \right)e^{\frac{-n\left(n+i\right)}{2}}\times B\left(t-t_{0}\right)$$

where $P_{n}\left(\cos\theta \right)$ are Legendre polynomials.

**Figure 4:**
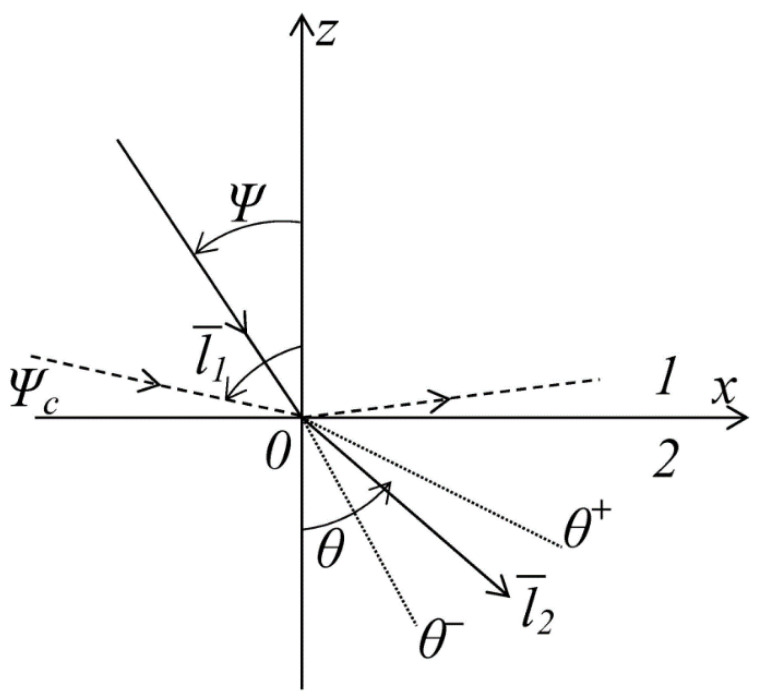
Diagram determining the direction of the crack propagation.

Using the probability densities (54), it is possible to solve the problem of determining the direction of crack propagation in Medium 2 near the interface with Medium 1 (Figure 4).

In a general case, the problem is formulated, as follows: to find the probability that the angle *θ* is located between the values of *θ^+^* and *θ^−^* at the time point *t*^1^:

(55) $$P\left(\theta^{-}<\theta <\theta^{+},t^{1}\right)=\int_{\theta^{-}}^{\theta^{+}}\theta f\left(\theta |t^{1}-t_{0},0\right)d\theta$$

The following formula calculates the probability that a crack will pass from Layer 1 into Layer 2 almost vertically:

(56) $$P\left(0<\theta <\xi ,t^{1}\right)=\int_{0}^{\xi }\theta f\left(\theta |t^{1}-t_{0},0\right)d\theta$$

where *ξ* is a small quantity.

In case, when a crack from Layer 1 approaches the interface at a small angle and, in fact, propagates along the interface between the layers, the probability is calculated by the formula:

(57) $$P\left(\frac{\pi }{2}-\xi <\theta <\frac{\pi }{2},t^{1}\right)=\int_{\frac{\pi }{2}-\xi }^{\frac{\pi }{2}}\theta f\left(\theta |t^{1}-t_{0},0\right)d\theta$$

Of interest is the most probable angle of the direction of crack propagation from Medium 1 into Medium 2 at the time point $\;t=t^{1}$, to be found by the formula:

(58) $$F\left(\theta \right)=\int_{0}^{\theta }f\left(\theta |t^{1}-t_{0},0\right)d\theta$$

The necessary condition for the minimum probability (58) has the form, from which the most probable θ* can be found:

(59) $$\frac{dF\left(\theta \right)}{d\theta }=0$$

The sufficient condition for the minimum is as follows:

(60) $$\frac{d^{2}F\left(\theta \right)}{d\theta^{2}}<0$$

In the case a crack stops at the boundary at the time point *t*_0_, and then at the random time point t* it passes from Layer 1 into Layer 2, it is possible to formulate the problem of finding the most probable time for the crack to pass from Layer 1 into Layer 2 and the most probable angle at this time moment.

In this case, a two-dimensional probability distribution function can be introduced through the formula, as follows:

(61) $$F\left(\theta ,t\right)=\int_{0}^{t}\int_{0}^{\theta }f\left(\theta |t^{1}-t_{0},0\right)d\theta dt$$

The necessary condition is

(62) $$\frac{\partial F}{\partial t}=0,\;\;\;\frac{\partial F}{\partial \theta }=0$$

The most probable θ* and *t*_1_ are to be found from (62). The sufficient condition: the matrix of

(63) $$\left|\begin{array}{cc} F_{,tt} & F_{,t\theta }\\ F_{,\theta t} & F_{,\theta \theta } \end{array}\right|$$

should be positively definite.

Let us consider the expression for *B* in (54). *B* can be found in the form of:

(64) $$B=\frac{n_{1}}{n_{2}}\sin\Psi$$

Thus, through *B* in (54), the probabilities depend on the physical and mechanical characteristics of Layer 1 (through *n*_1_) and Layer 2 (through *n*_2_) from the angle *Ψ*, under which the crack tip in Layer 1 approaches the interface with Layer 2.

## 4. Discussion

**Figure 5:**
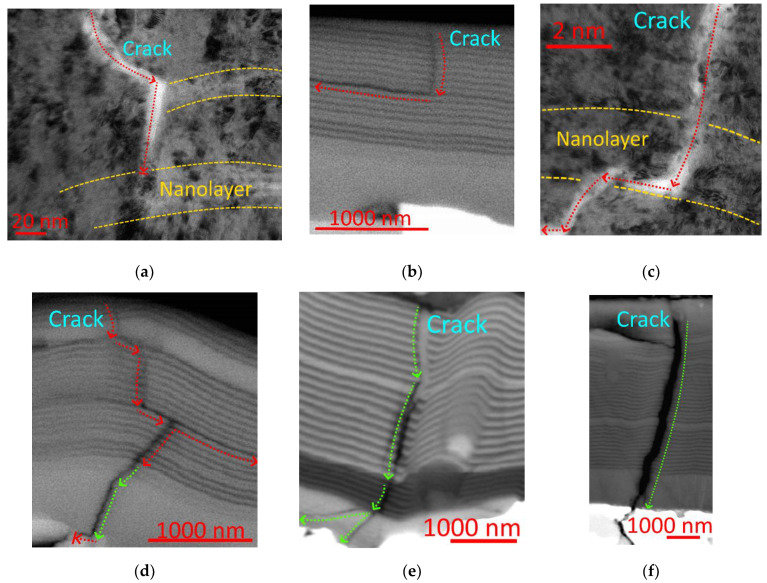
Examples of the crack propagation in the multilayer coating Ti-TiN-(Ti,Cr,Al)N (**b**,**d**–**f**) and Zr,Nb-(Zr,Nb)N-(Zr,Nb,Al)N (**a**,**c**): (**a**)—deceleration of the crack development in the nanolayer structure (transmission electron microscope (TEM)), (**b**)—transformation of the crack into delamination (scanning electron microscope (SEM)), (**c**)—deceleration of the crack development due to transformation into delamination between nanolayers (TEM), (**d**)—complex development of the crack with partial transformation into delamination (SEM), (**e**)—penetration by the crack through the coating structure with a slight deviation at the nanolayer interface (SEM), and (**f**)—penetration by the crack through the coating as a whole, with no noticeable influence of the nanolayer structure (SEM). The arrows indicate the direction of crack development.

On the qualitative level, the proposed model can be confirmed by the observation of the crack formation process in multilayer and nanolayer coatings during the action of stochastic loading. In particular, the cutting process is one of such processes [59]. Under the influence of loads (cutting forces) that arise during the process of cutting, microcracks are formed in the coating structure. In [2,3,21,35,36,52,53], it is found that the multilayer, in particular, nanolayer structure of the coating significantly affects the crack propagation. While, within one nanolayer, a crack usually propagates without changing its direction (in most cases, perpendicular to the nanolayer boundaries), then, while passing an interface between two nanolayers, a crack can either completely stop (Figure 5a) or transform into delamination between nanolayers (Figure 5b–d). Such a transformation can stop the crack propagation (Figure 5b) or form a step (Figure 5c,d). At the same time, the delamination can re-transform into a transverse crack (Figure 5c) or form a transverse crack branching from the delamination (Figure 5d). Under other conditions, a transverse crack can cut through the nanolayer structure without forming delaminations and slightly changing its direction at the nanolayer interface (Figure 5e), or with forming a straight transverse crack, which cuts through the coating as a whole (Figure 5f).

**Figure 6:**
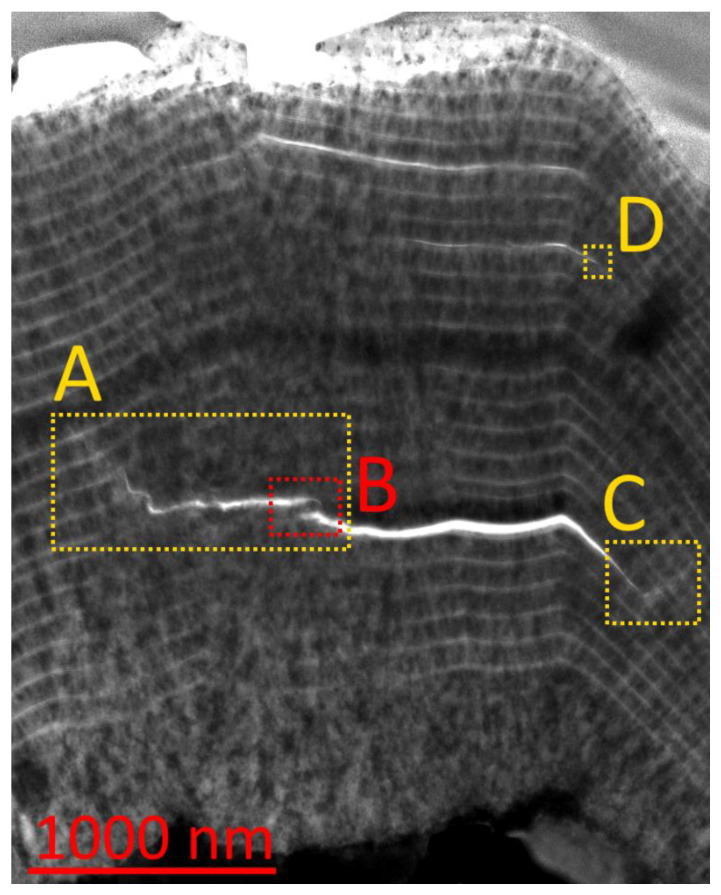
Formation of cracks and delaminations in the structure of the Zr,Nb-(Zr,Nb)N-(Zr,Nb,Al)N coating under the influence of the cutting conditions.

Let us consider the influence of the nanolayer coating structure on the pattern cracking using the example of the Zr,Nb-(Zr,Nb)N-(Zr,Nb,Al)N coating (Figure 6). In this coating, the nanolayers are characterized by a balanced combination of considerable hardness and ductility [57,58,59]. Figure 6 exhibits the structure of the coating that was deposited on a carbide substrate after the turning of steel. Cracks and internanolayer delaminations were formed in the coating structure as a result of the stochastic action of the cutting forces. An extensive delamination transforming into a transverse crack (Areas A, B, and C) arose in the internal area of the coating.

**Figure 7:**
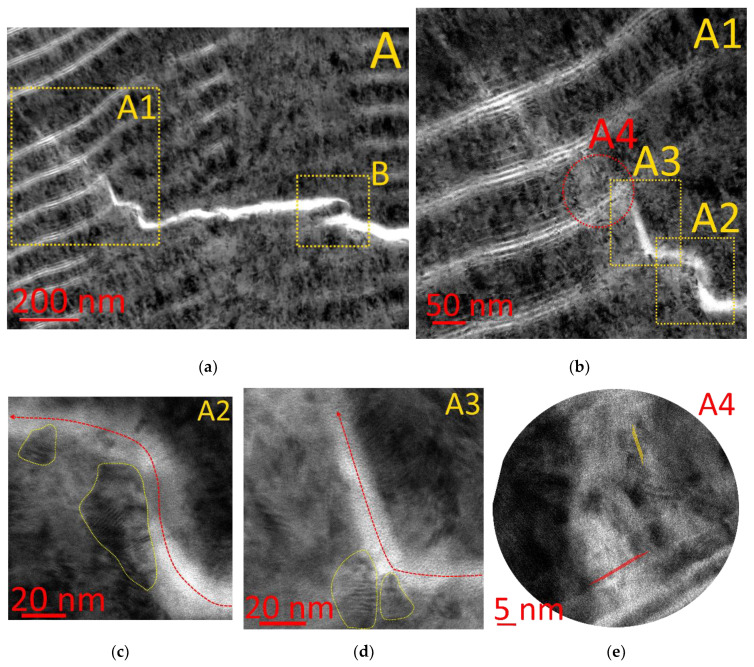
Pattern of crack propagation through the structure of the nanolayer coating. (**a**) general view of Area A, (**b**) General view of area A1—crack completion, (**c**,**d**) areas A2 and A3—fracture direction transformation, (**e**) Area A4—change in crystal structure during crack propagation.

Area A is of particular interest (Figure 7a), which is characterized by the transformation of the internanolayer delamination first into an inclined and then into a transverse crack. All three possible ways of crack propagation through the nanolayer interface are exhibited here: the transformation into delamination ($\Psi =\Psi_{c}$), the change of propagation direction (Ψ≠θ), and the retardation (P2 = 0, P1 = P11, T = 0, condition (45)). Let us consider Area A1 with the multiple transformation of the transverse crack into the delamination and reverse transformation. This process continues until the deformation energy decreases to a value, when no new transformation occurs and the crack growth stops [31]. At the same time, a decrease in power can be noticed in accordance with Equation (45). If the conditions (47) and (50) are met, then the energy is localized near the layer interface, which leads to the transformation of the crack into delamination (see Area A2, Figure 7c). A reverse transformation of the delamination into a transverse crack is possible (see Area A3, Figure 7c). In this case, condition (49) is met. It should be noted that the layer material was assumed to be isomorphic during the modeling. In reality, the layer material is neither isomorphic nor isotropic, and the crystalline structure of the layer influences the crack propagation. In particular, Area A2 (Figure 7c) depicts crystalline formations, which influence the crack growth direction. A crack can grow along the crystalline boundaries or can break a crystal (see Area A4, Figure 7e).

**Figure 8:**
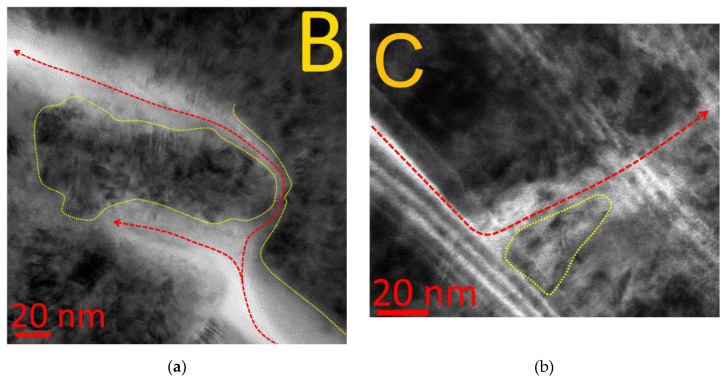
Pattern of crack propagation through the structure of the nanolayer coating. (**a**) Area B—crack division, (**b**) Area C—fracture direction transformation.

In some cases, the pattern of crack propagation upon reaching the layer interface can be more complex and, in particular, a crack can branch. For example, in Area B (Figure 8a), the crack branches into an internanolayer delamination (which stops about 100 nm from the branch point) and a transverse crack, which, then, in turn, transforms into an interlayer delamination. In Area C (Figure 8b), the interlayer delamination transforms into a transverse crack, which then cuts through two interlayer interfaces without changing its direction (the condition (49)), and then stops at the third interlayer interface (P2 = 0, P1 = P11, T = 0, the condition (45)).

**Figure 9:**
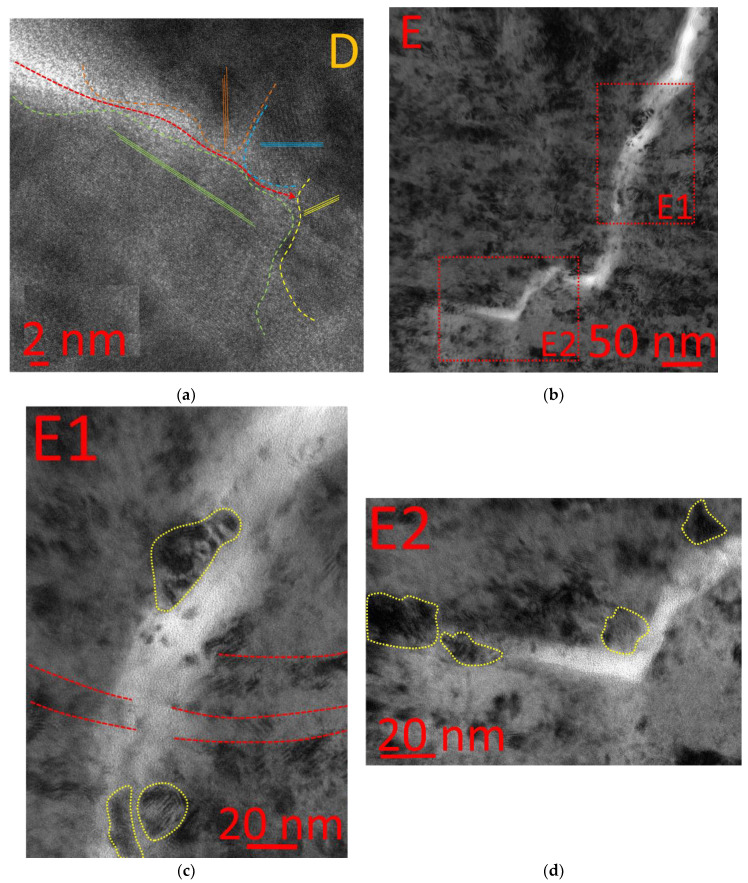
Pattern of crack Propagation through the structure of the nanolayer coating. (**a**) general view of Area D (**b**) general view of Area E, (**c**) Area E1, (**d**) Area E2.

The area of crack retardation (Area D, Figure 9a) is also of certain interest. It can be noticed how the crack retards gradually, moves along the crystal boundaries, and then stops, resting against the crystal boundary. At this moment, the crack propagation energy is insufficient to break the crystal or change the crack growth direction, and the crack propagation stops.

Similar patterns of crack propagation can be noticed in another area of the coating under consideration (Area E, Figure 9b–d). In particular, there is a step-by-step transformation of the crack growth direction under the mechanism of “delamination–transverse crack cutting through a nanolayer–delamination” (Figure 9b). The closer examination reveals the influence of the crystalline structure of the layers on the crack growth direction. With sufficient growth energy, the crack continues to grow along the intercrystalline interfaces and then cuts through the internanolayer interfaces (Area E1, Figure 9c) and, when the growth energy decreases, the crack stops, resting against the crystal at the nanolayer interface (Area E2, Figure 9d).

## 5. Conclusions

Thus, it can be noticed that, with a certain combination of properties of alternating nanolayers, it is possible to decelerate the crack development and, thus, increase the crack resistance and resistance to brittle fracture of the coating. The possibility of finding the most probable angle of the direction of the crack propagation from Medium 1 into Medium 2 while using the proposed technique makes it possible to predict the total resistance of a multilayer coating to brittle fracture, since the nanolayer coating usually consists of a repeating sequence of nanolayers that are identical in composition and thickness. It should be noted that the proposed model is dedicated to specific material of coating-solid ceramic materials based on nitrates, carbides, oxides, and carbonitrides of metals.

Thus, the proposed model includes the following stages of predicting:

- the determination of the crack propagation velocity in a layer by calculating the conditional probability for location of a crack tip within the interval of (l,l+Δl) at the time point *t*, if at the time point *t*_0_, the crack tip stays at the point *l*_0_. In this case, the theory of Markov processes is used in order to describe the crack formation process. This model also takes the surface roughness of the coating layer under consideration into account.
- Predicting the behavior of a crack when it passes through the layer interface based on predicting changes in the direction of the energy density vector. The presented modeling uses the optical-mechanical analogy, and the problem of determining the direction of crack propagation in Medium 2 near the interface with Medium 1 is to be solved by determining the probability densities. The probabilities that are to be determined depend on the physical and mechanical characteristics of Layers 1 and 2 and the angle *Ψ*, at which the crack tip in Layer 1 approaches the interface with Layer 2.
- It can be noticed that, in addition to the nanolayer structure, the pattern of crack propagation can also be affected by the crystalline structure of the coating. With a decrease in the deformation energy, the intercrystalline interfaces have a greater influence on the crack growth direction, and the crack can stop, resting against a crystal boundary. Thus, during the further modeling, it is also important to take the influence of the crystalline structure of the nanolayers into account.

In particular, as a possible direction for the further development of this model, the factors with a significant influence on crack formation in a multilayer coating can be considered, as follows:

- the layer interface is gradient, which is, the transition from one layer to another occurs smoothly with a gradual increase in the percentage composition of some elements and a decrease in others [21,35,36];
- a three-dimensional model more accurately reflects the specific features of the process, although it requires much more computational capabilities;
- the temperature factor affects both the mechanical properties of the layer materials and the state of the layer interfaces (in particular, due to the difference in the coefficients of thermal expansion of conjugate layers); and,
- internal and residual stresses (depending, in particular, on the total coating thickness and the thicknesses of nanolayers [59]) have a noticeable influence on the crack formation.

## Data Availability

The data presented in this study are available on request from the corresponding author. The data are not publicly available due to privacy.

## Abbreviations

*ω*	oscillation frequency in a layer under the action of cyclic loads
*G*	shear modulus
*E*	Young’s modulus
*ν*	Poisson’s ratio
*σ*_0_	yield strength
